# HPLC-DAD phenolics analysis, α-glucosidase, α-amylase inhibitory, molecular docking and nutritional profiles of *Persicaria hydropiper* L.

**DOI:** 10.1186/s12906-022-03510-7

**Published:** 2022-01-27

**Authors:** Mater H. Mahnashi, Yahya S. Alqahtani, Bandar A. Alyami, Ali O. Alqarni, Sultan A. Alqahl, Farhat Ullah, Abdul Sadiq, Alam Zeb, Mehreen Ghufran, Alexey Kuraev, Asif Nawaz, Muhammad Ayaz

**Affiliations:** 1 Department of Pharmaceutical Chemistry, College of Pharmacy, Najran University, Najran, Kingdom of Saudi Arabia; 2 Ahad Almsarha Hospital, Ahad Almsarha, Jazan Saudi Arabia; 3 Department of Pharmacy, Faculty of Biological Sciences, University of Malakand, Chakdara, Dir (L), KP 18000 Pakistan; 4 Department of Biochemistry, University of Malakand, Chakdara, Dir (L), KP 18000 Pakistan; 5 Department of Pathology, MTI Bacha Khan Medical College, Mardan, Pakistan; 6 K.G. Razumovsky Moscow State University of Technologies and Management (The First Cossack University), 73, Zemlyanoy Val St, Moscow, Russian Federation 109004

**Keywords:** HPLC-DAD analysis, Phenolics, *P. hydropiper*, Diabetes, Saponins, Molecular docking

## Abstract

**Background:**

Natural phenolic compounds and Phenolics-rich medicinal plants are also of great interest in the management of diabetes. The current study was aimed to analyze phenolics in *P. hydropiepr* L extracts via HPLC-DAD analysis and assess their anti-diabetic potentials using in-vitro and in-silico approaches.

**Methods:**

Plant crude methanolic extract (Ph.Cme) was evaluated for the presence of phenolic compounds using HPLC-DAD analysis. Subsequently, samples including crude (Ph.Cr), hexane (Ph.Hex), chloroform (Ph.Chf), ethyl acetate (Ph.EtAc), butanol (Ph.Bt), aqueous (Ph.Aq) and saponins (Ph.Sp) were tested for α-glucsidase and α-amylase inhibitory potentials and identified compounds were docked against these target enzymes using Molecular Operating Environment (MOE) software. Fractions were also analyzed for the nutritional contents and acute toxicity was performed in animals.

**Results:**

In HPLC-DAD analysis of Ph.Cme, 24 compounds were indentfied and quantified. Among these, Kaemferol-3-(p-coumaroyl-diglucoside)-7-glucoside (275.4 mg g^− 1^), p-Coumaroylhexose-4-hexoside (96.5 mg g^− 1^), Quercetin-3-glucoronide (76.0 mg g^− 1^), 4-Caffeoylquinic acid (58.1 mg g^− 1^), Quercetin (57.9 mg g^− 1^), 5,7,3′-Trihydroxy-3,6,4′,5′-tetramethoxyflavone (55.5 mg g^− 1^), 5-Feruloylquinic acid (45.8 mg g^− 1^), Cyanidin-3-glucoside (26.8 mg g^− 1^), Delphinidin-3-glucoside (24 mg g^− 1^), Quercetin-3-hexoside (20.7 mg g^− 1^) were highly abundant compounds. In α-glucosidase inhibition assay, Ph.Sp were most effective with IC_50_ value of 100 μg mL-1. Likewise in α-amylase inhibition assay, Ph.Chf, Ph.Sp and Ph.Cme were most potent fractions displayed IC_50_ values of 90, 100 and 200 μg mL-1 respectively. Docking with the α-glucosidase enzyme revealed top ranked conformations for majority of the compounds with Kaemferol-3-(p-coumaroyl-diglucoside)-7-glucoside as the most active compound with docking score of − 19.80899, forming 14 hydrogen bonds, two pi-H and two pi-pi linkages with the Tyr 71, Phe 158, Phe 177, Gln 181, Arg 212, Asp 214, Glu 276, Phe 300, Val 303, Tyr 344, Asp 349, Gln 350, Arg 439, and Asp 408 residues of the enzyme. Likewise, docking with α-amylase revealed that most of the compounds are well accommodated in the active site residues (Trp 59, Tyr 62, Thr 163, Leu 165, Arg 195, Asp 197, Glu 240, Asp 300, His 305, Asp 356) of the enzyme and Cyanidin-3-rutinoside displayed most active compound with docking score of − 15.03757.

**Conclusions:**

Phytochemical studies revealed the presence of highly valuable phenolic compounds, which might be responsible for the anti-diabetic potentials of the plant samples.

## Background

Diabetes mellitus (DM) is a chronic metabolic disorder of glucose processing and characterized by hyperglycemia. DM occur as a result of some abnormalities in insulin production, secretion or its action, dysfunction in carbohydrate, protein and fat metabolism and other complications [1, 2]. This state of hyperglycemia produces classical symptoms of polyuria, polydipsia and polyphagia [3]. Globally, it has been estimated that the occurrence of diabetes has increased, from 4% in 1995 to 5.4% by the year 2025 [4]. About 450 million peoples have been effected by DM worldwide and its prevalence is expected to increase 690 million by 2044 [5]. Diabetes is one of the most challenging serious metabolic disorder and is the leading cause of death worldwide. Long term high level of glucose can result in number of acute or chronic complications [6], and failure of various organs such as eyes, kidneys, liver, nerves, heart, and blood vessels [7]. Type 1 and Type 2 are two prominent types of DM [8]. Type-1 diabetes is associated with auto-immune destruction of pancreatic β-cells and characterized by absolute deficiency of insulin secretion [9]. Whereas, Type-2 diabetes accounts for 90% of cases and is caused by resistance of tissues to insulin action and decrease insulin secretion [10]. Type 2 diabetes can be prevented by managing obesity, diet control and with anti-diabetic drugs [11]. Regarding drug development against type-2 diabetes, one of the most important strategy is inhibition of enzymes implicated in glucose absorption from gastrointestinal tract. For instance, α-amylase and α-glucosidase enzymes are responsible for the breakdown of starch and oligosaccharides to glucose and their inhibition play a significant role to decrease the absorption of glucose in the intestine [12]. Consequently, inhibitors of these enzymes are the potential targets in the development of anti-diabetic drugs.

Since ancient times, medicinal plants and natural products have been employed as sources of medicine for the treatment of diabetes and alleviating human suffering mostly in developing countries [13–15]. More than 400 traditional plants have been reported for DM treatment, but only few of these have received scientific and medical evaluation to assess their efficacy [16]. Natural products such as galegine, andrographolide, and acarbose are used for type-2 diabetes treatment. Plant containing polyphenols have been reported to inhibit α–amylase and α–glucosidase enzymes associated with type 2 diabetes and to exhibit insulin like activities in the utilization of glucose [17]. Phenolic phytochemicals are secondary metabolites of plant origin, possess preventive management of various chronic diseases linked with oxidation such as diabetes and cardiovascular disease [18]. Large number of α-amylase and α-glucosidase inhibitors are produced by different microorganisms and plants to regulate the activities of these enzymes [19]. α-amylase inhibitors decrease the hyperglycemia that usually occur after eating meal by reducing the speed of starch conversion into glucose. Hence low alpha amylase level is needed in diabetic patients for keeping their sugar level under control.

Family Polygonaceae also known as knotwood or smartweed family, consist of 59 genera and 1300 species which are distributed worldwide [20]. *Polygonum, Persicaria, Coccoloba, Calligonum, Rumex* and *Rheum* are the largest genera of Polygonaceae family. Traditionally numerous species of this family are used in folk medicine and as vegetables [21]. The *Persicaria* genus having 100 species, is found throughout the world, plays a vital role as alternative medicines. *Persicaria hydropiper* L. also known as water-pepper, belonging to Polygonaceae family, that can be search out in South East Asia. The medicinal uses of *P. hydropiper* has been reported in epilepsy, inflammation, edema, rheumatoid arthritis, joint pain, headache, colic pain, fever and other infectious diseases. It can also be used as diuretic, central nervous system (CNS) stimulant, anthelmintic and in the treatment of hypertension, hemorrhoids, kidney diseases, diarrhea, bleeding, parasitic worms, piles and angina [22]. We reported the plant and some bioactive metabolites for neuroprotective [23–26], gastroprotective [27], antimicrobial [28] and cytotoxic potentials [29–31]. *P. hydropiper* contains flavonoids, chalcone derivatives, phenylpropanoid derivatives, phenolic compounds, antraquinon, isocumarine, terpenoids and steroids [20]. Among the phenolic compounds in the ethanolic extract of *P. hydropiper*, rutin has been reported for its anti-diabetic, antioxidant and anti-inflammation activity [32]. Apart from this, the anti-diabetic potential of the ethanolic extract of *P. hydropiper* leaves has also been reported in mice during oral glucose tolerance tests [33]. The current project was aimed to investigate the plant for detailed phenolic composition via HPLC-DAD analysis, evaluate its in-vitro and in-silico anti-diabetic as well as nutritional potentials.

## Materials and methods

### Plant material, extraction and fractionation

Several species being reported for efficacy in diabetes, the current plant *Persicaria hydropiper* (L.) Delarbre, F. Polygonaceae was selected for the study and was collected in consultation with botanical taxonomist (Dr. Gul Rahim) from a marshy area of Talash Dir Pakistan during the month of July, 2013. Whole study protocol on the selected plant complies with institutional, national and international guidelines for the use of plants. After identification by the taxonomist, a dried sample was deposited at the herbarium of University of Malakand, Chakdara (Dir), Pakistan with voucher (H.UOM.BG.107). After collection, plant was properly cleansed with distilled water and subjected to shade drying for about 30 days. Subsequently, the dried pant material was coarsely crushed with a cutter machine and resulted powder (4.5 kg) was transferred to stainless steel container and 22 L of 80% methanol was added for crude extraction purpose. Powder material was kept for about 15 days in the solvent with occasional shaking to fully remove any soluble constituents. Thereafter, solvent was removed, filtered and evaporated via a rotary evaporator (Heidolph Laborota 4000, Schwabach, Germany) [34]. Finally, we got about 290 g (6.44%) of crude methanolic extact (Ph.Cme). To get further sub-fractions, 250 g Ph.Cme was suspended in 500 ml of distilled water in a separating funnel and gradually washed with solvents (polarity directed) including Ph.Hex (3 × 500 ml), Ph.Chf (3 × 500 ml), Ph.EtAc (3 × 500 ml) Ph.Bt (3 × 500 ml) and H_2_O (3 × 500 ml). Lastly, we got 68 g (27.2%) of Ph.Hex, 27 g (10.8%) of Ph.Chf, 13 g (5.2%) of Ph.EtAc, 11 g (4.4%) of Ph.Bt and 37 g (14.8%) of Ph.Aq [35, 36]. These were stored in tight containers and kept at refrigerator temperature till further use.

### Extraction of saponins

For the isolation of crude saponins, about 60 g powder material was added to 100 ml of ethanol (20%) using a conical flask. The mixture was heated for 4 h via water bath (55 °C) with appropriated gradual shaking. Thereafter, the solvent was filtered and the powder material was again extracted with 200 ml of ethanol. The ethanol was combined and was placed in water bath until its volume was reduced to 40 ml. The fluid was transferred to separating funnel and with the subsequent addition of 20 ml diethyl ether. The mixture was shaked vigorously. Within the funnel two layers were formed, the diethyl ether and water. The diethyl ether layer was discarded and 60 mL of n-butanol was added to the aqueous layer. The resultant mixture was twice washed with 5% NaCl and finally the solvents were evaporated via water bath and 9 g of saponins residue was obtained [37, 38].

### HPLC -DAD analysis of Ph.Cme

For sample preparation, 100 mg extract was dissolved in 10 mL methanol (100%) and shaken for 1 h. Samples were filtered by syringe filter (PFTE filter, 0.45 μ, Agilent Technologies, Germany) in to HPLC vials (2 mL). Injection volume was 50 μL. Chromatographic analysis was performed following our previously reported standard procedure [39–41]. In brief, Agilent 1260 infinity HPLC system equipped with quaternary pump, degasser, auto-sampler and coupled with diode array detector was used for phenolics quantification of the test sample. Compounds separation was done via an Agilent rapid resolution Zorbax Eclipse plus C18 column with dimensions of 4.6 X 100 mm and 3.5 μm, and maintained at temperature of 25 °C and a flow rate of 1 ml min^− 1^. Chromatogram was obtained at 320 nm while absorption spectra was scanned at wide range of 200-600 nm and only higher purity peaks (95%) were quantified [39]. Phenolic compounds were identified by comparison of the retention time as well as absorption spectra with standards available analyzed simultaneously. Other compounds were identified via comparison of absorption spectra with published literature [42–44]. For unknown compounds, calibration curves of standards with same chromatographic response factor were used.

### In-vitro anti-diabetic studies

#### α-glucosidase inhibition assay

The inhibitory activity of our samples against α-glucosidase enzyme was evaluated using the established method of McCue et al. (2005) [45]. In brief, solutions of the α-glucosidase enzyme was prepared by dissolving 0.5 unit mL^− 1^ in a 0.1 M phosphate buffer (pH 6.9). The final enzyme solution contain 20 μl α-glucosidase (0.5 unit mL^− 1^) and 120 μl 0.1 M phosphate buffer. Substrate solution consisting of p-Nitrophenyl-α-D-glucopyranoside (5 mM) was prepared in the same buffer (pH 6.9). Test samples at concentration range of 31.25-1000 μg mL^− 1^ were prepared and were mixed with enzyme solution followed by incubation for 15 min at 37 °C. Finally, 20 μl substrate solution was added to the enzyme mixture and was again incubated for 15 min at 37 °C. The reaction was completed by the addition of 80 μl of 0.2 M sodium carbonate solution. Absorbance were measured at 405 nm using UV visible spectrophotometer (Thermo electron corporation USA). The system without α-glucosidase act as blank, and acarbose was used as positive control. Each experiment was conducted in triplicate and percent inhibition were calculated using formula;

$$\%\mathrm{inhibition}=\mathrm{Control}\ \mathrm{absorption}-\mathrm{Sample}\ \mathrm{absorption}/\mathrm{Control}\ \mathrm{absorption}$$

#### α-amylase inhibition assay

In-vitro amylase inhibition of our samples were performed according to the previously reported protocol [46]. Briefly, 100 μL of test samples were added to 200 μL of enzyme solution and 100 μL (2 mM) of phosphate buffer (pH -6.9). Thereafter, the mixture was incubated for 20 min and subsequently, 100 μL of 1% starch solution will be added to it. The same was repeated for the controls where 200 μL of the enzyme will replaced by buffer. After incubation for 5 min, 500 μL of dinitrosalicylic acid reagent was added to both control and test groups. Both samples were incubated for 10 min and absorbance’s were recorded at 580 nm via spectrophotometer. Percent inhibition were calculated using the formula;

$$\%\mathrm{inhibition}=\left[1\hbox{-} \left(\mathrm{A}/\mathrm{B}\right)\right]\times 100$$

Where A = absorbance of test and B = absorbance of enzyme control.

### Molecular docking with HPLC-DAD identified compounds

In-silico docking is an important tool to assess the mode of molecular interactions of new compounds within the target molecule as a potential inhibitor or activating agent [47]. The binding interactions of identified compounds in the active sites of our target enzymes α-glucosidase and α-amylase were elucidated via MOE-Dock software. The crystal structure of α-glucosidase is not available yet, so, we used homology model as described by Ming Liu et al [48] while the 3D crystal structure of the α-amylase (4 W93) was retrieved from the Protein Databank (PDB). Prior to molecular docking, all water molecules and ions were removed from the retrieved crystal structure using the Molecular Operating Environment software (www.chemcomp.com). The hydrogen atoms were added to the protein structures by 3D protonation and then energy minimization were carried out by using the default parameters of the MOE (gradient: 0.05, Force Field: Amber99).

The structures of the compounds were built in MOE and energy minimized using the default parameters of the MOE [49]. Both α-glucosidase and α-amylase were allowed to dock to the compounds using MOE by the default parameters i.e., Placement: Triangle Matcher, Refinement: Induced Fit, Rescoring: London dG. For each ligand ten conformations were generated. The top-ranked conformation of each compound was used for further analysis. After the molecular docking, the best poses having polar, arene-arene, H-pi and pi-H interactions were analyzed by Pymol software.

### Nutritional contents

#### Assessment of moisture content

Loss on drying (LOD) method was followed for the determination of moisture content of the plant sample. A weighed quantity of powdered plant sample was taken in a suitable container and allowed to dry at 105 °C in oven till the achievement of constant weight. Thus the amount of moisture present in the powdered plant sample was figured out from the difference of dried weight of sample and the total weight of the sample.

#### Assessment of ash content

Incineration procedure was followed for determination of ash content of powdered plant sample. A weighed amount of sample was put in a crucible and transferred into the muffle furnace and allowed to incinerate at 550 °C for 24 h. Similarly total ash content was figured out after conversion of dried mass of powdered plant sample into ashes.

#### Assessment of crude fat

Soxhlet method was followed for the determination of total fats in the sample. Briefly, 2 g of dried powdered plant sample was transferred into a soxhlet extractor and petroleum ether was added to the flask of the extractor. The extraction was carried out for 6 h till the exhaustion of sample from fat content. The obtained petroleum ether was filtered and the filtrate obtained was allowed to be evaporated in a weighed beaker. Similarly, the total fats were calculated as the total increase in weight of the beaker.

#### Assessment of crude protein

For determination of crude protein the method of microkjeldahl nitrogen method was followed. This method involved the digestion of plant sample with concentrated sulphuric acid and catalyst for the conversion of organic nitrogen into ammonium sulfate in the solution. After which the decomposition of ammonium sulfate was carried out via NaOH. The liberated ammonia was distilled into 5% boric acid. After this the titration of trapped ammonia was carried out with 0.05 N HCl for the deduction of nitrogen from ammonia. The indicators used were methylene red and blue both. The percent proteins were calculated from the value of nitrogen obtained multiplied by 6.25.

### Toxicity evaluations

#### Animals and ethical committee approval

BALB/c albino mice (18-35 g) mixed breed were used in the acute toxicity study. Animals were provided appropriate food and water ad libitum. Our study was evaluated and approved by Departmental Research Ethics Committee (DREC) via reference no DREC/2016052/01. Animals studies were performed following rules of Institute of Laboratory Animal Resources Commission on life sciences, National research council 1996 [50].

#### Acute toxicity study

Test samples were evaluated for acute toxicity in mice after oral administration of increasing doses up to 2000 mg kg^− 1^. Animals were observed for lethality and aberrant behavioral changes [51].

#### Haemagglutination study

Haemagglutination activity was performed pursuing the procedure followed by Naqvi et al. [52]. Blood taken from healthy individuals consisting of different groups was centrifuged and 2% suspension of RBCs of each blood group was prepared in phosphate buffer (pH 7). Serial dilutions of each plant sample were prepared and 1 ml of each dilution was combined with 1 ml of each RBCs suspension. The solutions were kept for a while in test tubes at 25 °C. Negative haemagglutination activity was shown by the formation of smooth button at the bottom of test tube while positive activity was indicated by the formation of rough granular deposition. The intensity of activity was measured by the extent of smooth button formation or deposition.

### Statistical analysis

All tests were performed in triplicate and results were presented as Mean ± SEM. Results were expressed as % inhibition (mean ± SEM of *n* = 3) and IC_50_. IC50 were calculated from dose-response curve along the doses tested in the inhibition studies. Values significantly different as compare to standard drug One way ANOVA followed by multiple comparison DUNNETT test was applied to the data for comparison with the standard group. *: *p* < 0.05, **: *p* < 0.01, ***: *p* < 0.001. ns: Results not significantly different in comparison to standard drug.

## Results and discussion

### HPLC-DAD phenolic-profiling

**Fig. 1:**
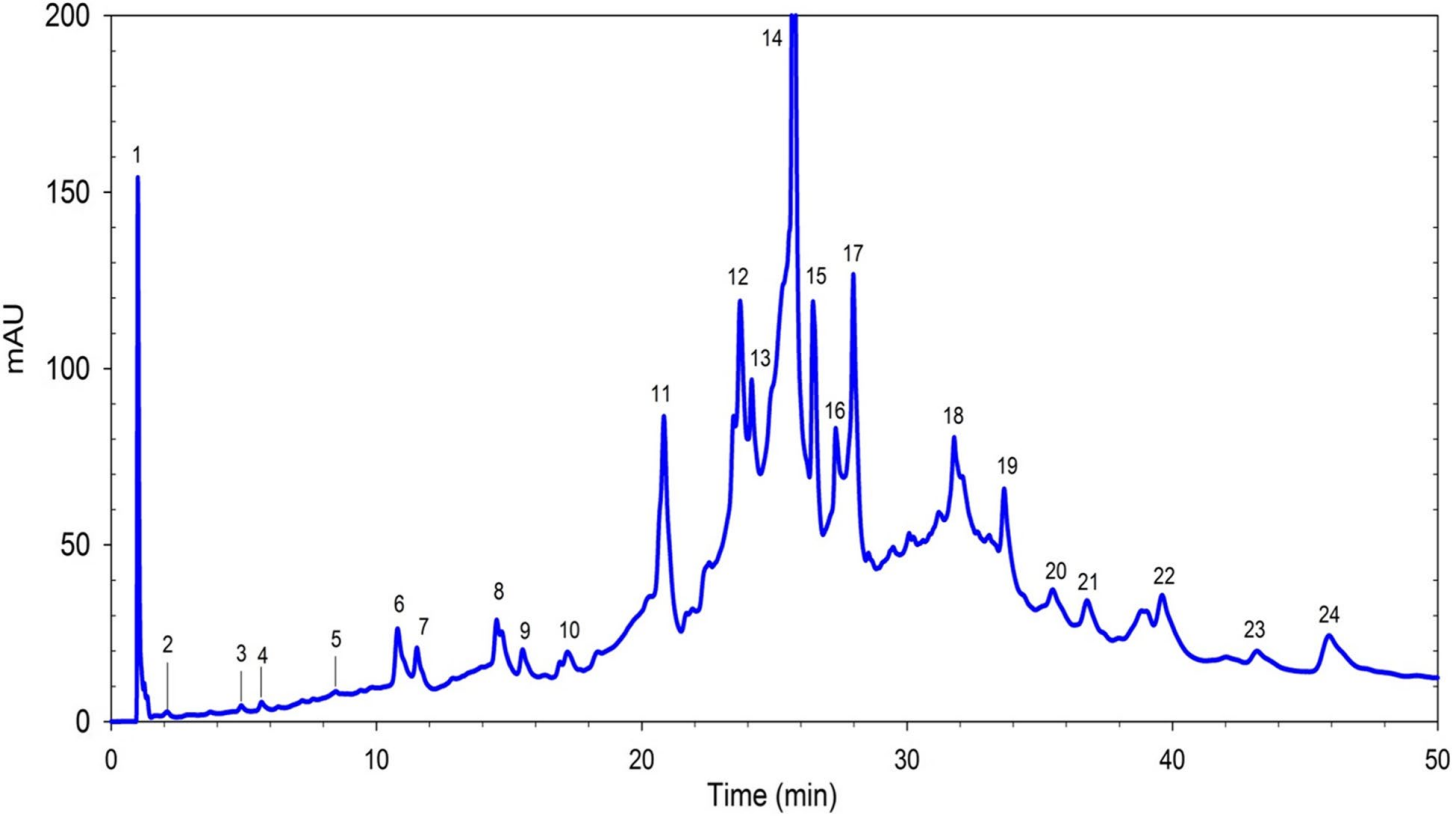
Chromatogram of the HPLC-DAD analysis of Ph.Cme. Peak numbers represent individual compounds and their details are provided in Table 1

HPLC-DAD analysis of Ph.Cme is summarized in Table 1 and Fig. 1. Chromatogram exhibit identification of 24 phhenolic compounds. The most abundant identified compounds were Kaemferol-3-(p-coumaroyl-diglucoside)-7-glucoside (275.4 mg/g peak 14), p-Coumaroylhexose-4-hexoside (96.5 mg/g peak 11) and Quercetin-3-glucoronide (76.0 mg/g peak 17). Other abundant compounds were 4-Caffeoylquinic acid (58.1 mg/g), Quercetin (57.9 mg/g), 5,7,3′-Trihydroxy-3,6,4′,5′-tetramethoxyflavone (55.5 mg/g) Ellagic acid (50.4 mg/g), 5-Feruloylquinic acid (45.8 mg/g), Cyanidin-3-glucoside (26.8 mg/g), Delphinidin-3-glucoside (24 mg/g), Quercetin-3-hexoside (20.7 mg/g), 5,7-dihydroxy-4′-methoxyflavone (15.2 mg/g) of the sample. Among the other compounds were Hydroxybenzoic acid (2.8 mg/g), Gallic acid (0.2 mg/g), Hydroxybenzoylhexose (0.2 mg/g), Caffeic acid (0.2 mg/g), Syringic Acid (0.2 mg/g), p-Coumaric acid (8.8 mg/g), 5-Coumaroylquinic acid (5.2 mg/g), 3-Caffeoylquinic acid (6.2 mg/g), 3-Coumaroylquinic Acid (3.8 mg/g), p-Coumaroylhexose (5.1 mg/g), Malvidin-3-glucoside (3.8 mg/g), Cyanidin-3-rutinoside (12.0 mg/g) respectively (Table 1, Fig. 1).

**Table 1:** Phenolic profile of Ph.Cme extract (mg/g)

Peak	Rt (min)	Identity	Mean Composition (mg/g) of sample	STD
1	1	Hydroxybenzoic acid	**2.8**	**0.02**
2	2.1	Gallic acid	**0.2**	**0.01**
3	4.9	Hydroxybenzoylhexose	**0.2**	**0.01**
4	5.6	Caffeic acid	**0.2**	**0.01**
5	8.4	Syringic Acid	**0.2**	**0.01**
6	10.7	p-Coumaric acid	**8.8**	**0.2**
7	11.5	5-Coumaroylquinic acid	**5.2**	**0.2**
8	14.5	3-Caffeoylquinic acid	**6.2**	**0.3**
9	15.5	3-Coumaroylquinic Acid	**3.8**	**0.1**
10	17.2	p-Coumaroylhexose	**5.1**	**0.1**
11	20.8	p-Coumaroylhexose-4-hexoside	**96.5**	**2.4**
12	23.7	4-Caffeoylquinic acid	**58.1**	**1.0**
13	24.1	5-Feruloylquinic acid	**45.8**	**1.0**
14	25.7	Kaemferol-3-(p-coumaroyl-diglucoside)-7-glucoside	**275.4**	**6.5**
15	26.4	Ellagic acid	**50.4**	**0.5**
16	27.3	Quercetin	**57.9**	**1.3**
17	27.9	Quercetin-3-glucoronide	**76.0**	**1.2**
18	31.6	5,7-dihydroxy-4′-methoxyflavone	**15.2**	**0.2**
19	33.6	5,7,3′-Trihydroxy-3,6,4′,5′-tetramethoxyflavone	**55.5**	**1.2**
20	35.4	Cyanidin-3-glucoside	**26.8**	**0.3**
21	36.7	Delphinidin-3-glucoside	**24.0**	**0.4**
22	39.6	Quercetin-3-hexoside	**20.7**	**0.8**
23	43.1	Malvidin-3-glucoside	**3.8**	**0.1**
24	45.9	Cyanidin-3-rutinoside	**12.0**	**0.2**

**Standard compounds used were;** Hydroxybenzoic acid, Gallic acid, Caffeic acid, Syringic acid, p-coumaric acid, 3-Caffeoylquinic acid, Quercetin, Ellagic acid and Cyanidin-3-glucoside

### Enzymes inhibition studies

Natural phenolics are widely known and scientifically validated for efficacy in DM. For instance, mulberry polyphenolic compounds such as syringic acid and galloylcyanidin-glycoside are reported to inhibit *α-*glucosidase activity while quercetin and cyanidin-glycosides are essential for cellular antioxidant activity [53]. Quercetin is reported to control glucose homeostasis of whole-body by interacting with various molecular targets in small intestine, pancreas, skeletal muscle, liver and adipose tissue. Quercetin mechanisms of action include intestinal glucose absorption inhibition, insulin-sensitizing and secreting activities and increased utilization of glucose in peripheral tissues [54]. Ellagic acid seems to play an anti-diabetic activity. The anti-diabetic effect of ellagic acid through the action on pancreas β-cells, decreasing glucose intolerance and stimulation of insulin secretion has been reported by Fatima et al., [55]. Likewise, fruit extract of *Emblica officinalis* exhibit anti-diabetic potentials via increased insulin sensitization preimirilymediated by the presence of gallic acid [56]. The antioxidant and anti-diabetic potential of caffeic acid in a streptozotocin-induced diabetic rat model has been evaluated which showed a significant increase in serum insulin level, and decrease glucose level in the blood of diabetic rat models [57]. It has also been demonstrated that cyanidin-3-O-glucoside inhibit glucosidase enzyme which result in decrease glucose absorption in intestine [58]. Anti-diabetic and antioxidant activity of sweet cherries [59] and *Prunus avium* [60] has been reported which may be due to the identified phenolic contents, including hydroxybenzoic acid. The hydroxybenzoic acid and *p*-coumaric acid are probably responsible for the anti-diabetic activity investigated in edible mushrooms by D.Stojkovic et al., 2019 [61]. Among the phenolic compounds in the ethanolic extract of *P. hydropiper*, rutin has been reported for its anti-diabetic, antioxidant and anti-inflammation activity [32]. The identified phenolics might contribute to the overall anti-diabetic potentials of our test samples.

In the present study, Ph.Sp was found highly active against α-glucosidase enzyme as shown in Table 2. Overall a concentration dependent inhibition was observed against the enzyme. Ph.Sp exhibited 71.50 ± 0.28% inhibitory activity at the high tested dose (1000 μg mL^− 1^). Acarbose inhibitory activity at the same dose was 77.30 ± 0.61%. The inhibitory activity of Ph.Sp was comparable to the standard drug acarbose at the same concentrations. The IC_50_ for Ph.Sp and acrabose were 100 and 18 μg/ml respectively. Among the other fractions, Ph.Cr, Ph.Hex, Ph.Chf, Ph.EtAc, Ph.Bt and Ph.Aq have displayed concentration dependent inhibitions with IC_50_ of 400, 1800, 320, 680, 1000 and 700 μg mL^− 1^ respectively. Ph.Cr, Ph.Chf and Ph.Sp are most active samples and need further in-vivo studies for potential effectiveness against type 2 DM. The Ph.Cr, Ph.Chf can be subjected to column chromatography for isolation of bioactive compounds.

**Table 2:** Results of α-glucosidase and α-amylase inhibitory potentials of *Persicaria hydropiper*

Samples	α-glucosidase assay								α-amylase assay				
	Conc.	% Inhibition	IC_50_ μg/mL	Sample	Conc.	% Inhibition	IC_50_ μg/mL	Sample	% Inhibition	IC_50_ μg/mL	Samples	% Inhibition	IC_50_ μg/mL
Ph.Cr	1000 500 250 125 62.5 31.25	67.33 ± 1.52* 51.86 ± 3.09*** 42.50 ± 1.32*** 35.20 ± 2.52*** 29.66 ± 2.56*** 25.50 ± 1.32***	400	Ph.Bt	1000 500 250 125 62.5 31.25	49.67 ± 1.52*** 43.00 ± 1.73*** 38.67 ± 0.57*** 32.00 ± 0.00*** 24.00 ± 0.00*** 19.20 ± 2.25***	1000	Ph.Cr	70.89 ± 0.55** 59.93 ± 1.45** 51.02 ± 0.78* 43.83 ± 0.38 ^ns^ 32.56 ± 2.12* 19.55 ± 1.56**	200	Ph.Bt	57.58 ± 0.15^ns^ 51.68 ± 2.33* 33.90 ± 1.22*** 20.17 ± 0.88*** 11.55 ± 2.50*** 05.40 ± 1.90***	550
Ph.Hex	1000 500 250 125 62.5 31.25	32.66 ± 2.52*** 26.16 ± 2.75*** 21.50 ± 1.32*** 17.73 ± 0.51*** 15.69 ± 1.04*** 11.93 ± 1.61***	1800	Ph.Aq	1000 500 250 125 62.5 31.25	53.83 ± 1.04*** 45.00 ± 1.00*** 41.86 ± 3.09*** 34.33 ± 2.02*** 30.36 ± 0.57** 22.00 ± 0.00**	700	Ph.Hex	55.78 ± 0.55*** 42.33 ± 1.10*** 29.00 ± 2.41*** 12.88 ± 0.91*** 07.96 ± 2.44*** 03.63 ± 1.91***	750	Ph.Aq	41.99 ± 2.40** 35.71 ± 3.20*** 26.21 ± 1.50*** 15.00 ± 0.62*** 10.94 ± 2.86** 4.99 ± 1.00***	920
Ph.Chf	1000 500 250 125 62.5 31.25	66.44 ± 1.50* 54.73 ± 0.51*** 47.23 ± 1.05*** 40.16 ± 1.02* 34.50 ± 1.32 ^ns^ 29.00 ± 0.00	320	Ph.Sp	1000 500 250 125 62.5 31.25	71.50 ± 0.28 ^ns^ 65.00 ± 0.86* 58.00 ± 0.28* 51.00 ± 1.15 ^ns^ 39.00 ± 0.00 ^ns^ 30.36 ± 0.57*	100	Ph.Chf	87.32 ± 2.45* 77.54 ± 0.87^ns^ 68.49 ± 1.33 ^ns^ 60.95 ± 2.40 ^ns^ 48.97 ± 0.51 ^ns^ 27.88 ± 1.20**	90	Ph.Sp	90.06 ± 0.45 ^ns^ 80.13 ± 1.77 ^ns^ 68.83 ± 0.64 ^ns^ 58.56 ± 0.84 ^ns^ 47.26 ± 2.35 ^ns^ 29.33 ± 1.68**	100
Ph.EtAc	1000 500 250 125 62.5 31.25	55.00 ± 1.00*** 43.60 ± 1.76*** 34.00 ± 1.00*** 28.20 ± 1.04*** 22.00 ± 0.00*** 17.00 ± 1.00***	680	P.C	1000 500 250 125 62.5 31.25	77.30 ± 0.61 73.00 ± 0.00 69.00 ± 0.00 55.50 ± 1.04 49.83 ± 0.44 41.00 ± 0.00	18	Ph.EtAc	68.00 ± 0.51^ns^ 49.60 ± 2.23* 37.95 ± 0.77** 21.40 ± 2.25** 15.86 ± 1.33*** 09.71 ± 2.55***	480	P.C	77.30 ± 0.61 73.00 ± 0.00 69.00 ± 0.00 55.50 ± 1.04 49.83 ± 0.44 41.00 ± 0.00	18

Results were expressed as % inhibition (mean ± SEM of *n* = 3) and IC_50_. Values significantly different as compare to standard drug (Acarbose), *: *p* < 0.05, **: *p* < 0.01, ***: *p* < 0.001. **ns:** Results not significantly different in comparison to standard drug. **P.C** = Acarbose

In amylase inhibition studies, all fractions displayed a concentration dependent inhibition of α-amylase enzyme with Ph.Sp and Ph.Chf with highest percent inhibitions. Ph.Sp and Ph.Chf exhibited 90.06 ± 0.45% and 87.32 ± 2.45 inhibitions at highest tested concentration (1000 μg mL^− 1^) respectively (Table 2). The IC_50_ for Ph.Sp and Ph.Chf were 100 and 90 μg mL^− 1^ respectively. Percent inhibitions of these fractions were very comparable with standard inhibitions. Among the other fractions Ph.Cr, Ph.Bt and Ph.EtAc showed moderate inhibitory activity with IC_50_ of 200, 550 and 480 μg mL^− 1^ respectively.

Natural products of enormous structural miscellany are still major source for the development of new drugs including inhibitors of glucose metabolizing enzymes [62, 63]. α-glucosidase inhibitors (AGI’s), like acarbose, voglibose in microorganisms and nojirimycin, 1-deoxynojirimycin has been reported from plants [64–66]. Commercially accessible AGI’s for instance acarbose, miglitol and voglibose are widely employed for the treatment of type 2 DM. These AGI’s are shown to diminish the insulin requirements for type 1 diabetes as well as improves reactive hypoglycemia [67]. As the AGI’s show therapeutic effect by restraining carbohydrate absorption, the undigested carbohydrate dislocate to the colon go through fermentation by colonic flora to result in adverse effects such as flatulence, abdominal discomfort and diarrhea [68]. But the undesirable effects are dose dependent and diminishes with the duration of therapy [69]. Recently, numerous efforts have been made to find out more effective drugs against type 2 diabetes from natural sources to develop physiologic functional food or isolate new and more effective compounds [70]. Several AGI present as phyto-constituents including alkaloids, glycosides, flavonoids, terpenoids and phenolic compounds have been reported from plant origin [71]. Thus, there is an urgent need to search for novel drugs from several sources, including natural products, with increased potency and lesser adverse effects than the existing drugs to fight global health problems posed by DM.

### Docking analysis of α-glucosidase

**Fig. 2:**
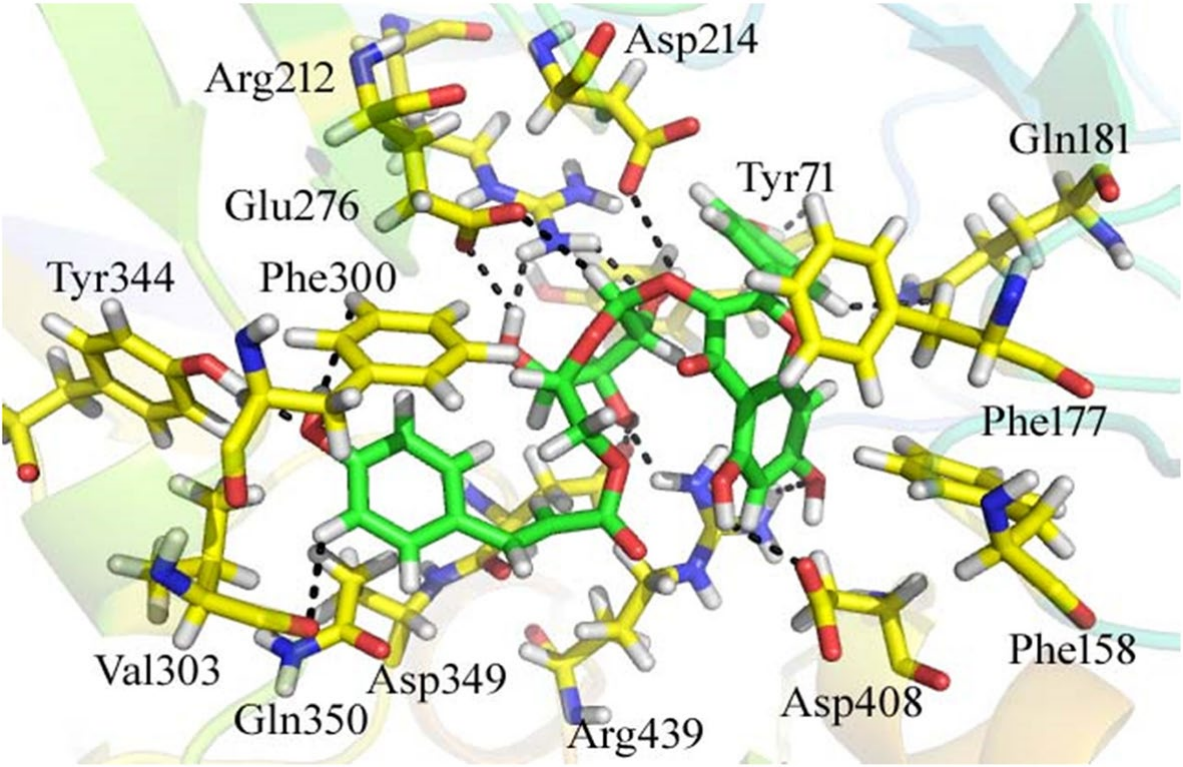
Molecular docking conformations of compound **14** against *α*-glucosidase

The docking results of the compounds with the alpha glucosidase enzyme have given good information about the nature of the binding mode. Our current docking findings revealed that majority of the compounds exhibited good confirmations in alpha glucosidase enzyme and were involved in various type of interactions with the active site residues of the target enzymes. The detail of docking scores and interactions for all compounds are listed in Table 3. From the docking conformation of the compounds, it was revealed that the top most active compound was compound 14 (docking score = − 19.80899) formed 14 hydrogen bonds, two pi-H and two pi-pi linkages with the Tyr 71, Phe 158, Phe 177, Gln 181, Arg 212, Asp 214, Glu 276, Phe 300, Val 303, Tyr 344, Asp 349, Gln 350, Arg 439, and Asp 408 residues of the binding pocket of the α-glucosidase as shown in Fig. 2. The high potency of the ligand might be due to the presence of the electron donating group (−OH) as well as the electron cloud system of the compound.

**Table 3:** Results of molecular docking studies with the identified compounds against *α*-glucosidase

S. No	Ligand		Receptor			Interaction	Distance	E (kcal/mol)	Docking score
1	O	14	ND2	ASN	347	H-acceptor	3.29	−2.9	−7.43843651
	O	15	ND2	ASN	347	H-acceptor	3.02	−2.4	
2	O	9	OE2	GLU	276	H-donor	2.58	−1.2	−11.3185654
	O	13	O	ASP	349	H-donor	2.77	−1.9	
	O	18	NH1	ARG	439	H-acceptor	2.74	−3	
3	O	33	OE1	GLN	350	H-donor	3.04	−0.6	−9.3436832
	O	24	ND2	ASN	347	H-acceptor	3.24	−1.3	
	O	24	6-ring	PHE	300	H-pi	3.36	−0.5	
4	O	12	OD2	ASP	408	H-donor	2.99	−2	−10.572752
	O	19	NE2	HIS	111	H-acceptor	3.27	−3.5	
	O	20	NH1	ARG	212	H-acceptor	3.29	− 2.9	
	O	20	NH2	ARG	212	H-acceptor	3.15	−2.1	
	O	20	NH1	ARG	212	ionic	3.29	−2.8	
	O	20	NH2	ARG	212	ionic	3.15	−3.6	
5	O	14	ND2	ASN	347	H-acceptor	2.93	−3.4	−8.25566006
	C	16	6-ring	PHE	300	H-pi	3.99	−0.5	
6	O	18	NE2	HIS	348	H-acceptor	2.99	−0.8	−7.69557381
7	O	18	OE1	GLN	350	H-donor	3.36	−0.6	−9.5489674
	O	22	OE1	GLN	350	H-donor	3.06	−2.5	
	O	41	5-ring	HIS	279	H-pi	3.6	− 2.6	
8	C	5	O	ASP	349	H-donor	3.21	−0.1	−18.5305271
	O	17	OE1	GLN	350	H-donor	2.77	−2	
	C	43	OD2	ASP	349	H-donor	3.78	−0.1	
	C	45	OE1	GLU	276	H-donor	3.86	−0.1	
	C	52	OD2	ASP	68	H-donor	3.21	−0.2	
	O	56	OD2	ASP	68	H-donor	2.79	−6.8	
	O	19	CD2	PHE	300	H-acceptor	3.17	−0.1	
	O	41	ND2	ASN	347	H-acceptor	3.35	−0.1	
	O	16	NH2	ARG	312	ionic	2.76	−6.3	
	−6	ring	CZ	PHE	177	pi-H	4.24	−0.1	
	−6	ring	NH1	ARG	439	pi-cation	4.27	−0.4	
9	C	26	OD1	ASP	214	H-donor	3.52	−0.2	−13.9830008
	C	30	OD1	ASP	214	H-donor	3.7	−0.1	
	O	35	OD2	ASP	349	H-donor	2.89	−3.7	
	O	40	OE1	GLN	181	H-donor	3.57	−0.1	
	O	35	NH1	ARG	439	H-acceptor	2.86	−0.3	
	O	38	NE2	HIS	348	H-acceptor	2.78	−5.9	
	O	39	NE2	HIS	348	H-acceptor	2.89	−2.1	
	O	40	NE2	HIS	111	H-acceptor	3.1	−1.6	
	O	38	NH1	ARG	212	ionic	3.25	−3	
	O	38	NH2	ARG	212	ionic	2.91	−5.1	
	O	39	NH2	ARG	212	ionic	3.31	− 2.7	
	C	26	6-ring	TYR	71	H-pi	4.79	−0.1	
	O	33	6-ring	PHE	177	H-pi	3.06	−0.2	
10	C	5	O	ASP	349	H-donor	3.34	−0.4	−13.088253
	O	22	OE1	GLN	350	H-donor	3.06	−0.2	
	C	32	OD2	ASP	68	H-donor	3.16	−0.2	
	O	40	OE1	GLN	181	H-donor	2.72	−1.6	
	O	22	CG2	VAL	303	H-acceptor	3.26	−0.1	
	C	35	6-ring	PHE	177	H-pi	3.49	−0.1	
	−6	ring	NE2	HIS	111	pi-H	3.91	−0.1	
11	C	26	O	PHE	157	H-donor	3.55	−0.1	−13.8564711
	C	41	OD2	ASP	408	H-donor	3.37	−0.3	
	O	21	CE1	PHE	177	H-acceptor	3.78	−0.1	
	O	35	N	ARG	312	H-acceptor	3.4	−0.3	
	O	45	CB	ARG	312	H-acceptor	3.64	−0.1	
	O	45	NE	ARG	312	H-acceptor	3.12	−1.9	
	O	64	CD1	PHE	158	H-acceptor	3.78	−0.1	
	O	64	CE1	PHE	158	H-acceptor	3.8	−0.1	
	O	64	CD1	PHE	177	H-acceptor	3.26	−0.1	
	C	1	6-ring	PHE	157	H-pi	4.44	−0.1	
	C	3	6-ring	PHE	157	H-pi	4.44	−0.1	
	−6	ring	NH1	ARG	439	pi-cation	3.14	−0.1	
12	C	4	OD1	ASP	214	H-donor	3.49	−0.1	−15.2827396
	O	10	OE1	GLN	181	H-donor	2.78	−2.4	
	C	23	O	ASP	349	H-donor	3.61	−0.1	
	O	20	NH2	ARG	212	H-acceptor	3.15	−0.8	
	O	20	CZ	PHE	300	H-acceptor	3.89	−0.1	
	O	39	NE	ARG	312	H-acceptor	2.92	−0.9	
	O	39	NH2	ARG	312	H-acceptor	2.94	−0.5	
	O	40	CD	ARG	312	H-acceptor	3.22	−0.1	
	O	39	NE	ARG	312	ionic	2.92	−5.1	
	O	39	NH2	ARG	312	ionic	2.94	−4.9	
	O	40	NE	ARG	312	ionic	2.97	−4.7	
	−6	ring	NE2	HIS	111	pi-H	4.84	−0.1	
13	C	3	OD1	ASP	214	H-donor	3.16	− 0.3	−12.4345493
	O	16	OD1	ASP	214	H-donor	2.8	−5.3	
	O	42	OE2	GLU	304	H-donor	2.75	−3.7	
	O	42	CD2	PHE	300	H-acceptor	3.68	−0.1	
	O	42	CD	ARG	312	H-acceptor	3.1	−0.1	
	O	44	NE2	HIS	245	H-acceptor	2.91	−7.1	
	O	44	CD2	HIS	279	H-acceptor	3.23	−0.3	
	O	45	CD2	LEU	218	H-acceptor	4.03	−0.1	
	O	45	NE2	HIS	245	H-acceptor	3.38	−1.1	
	O	39	6-ring	PHE	157	H-pi	4.32	−0.4	
14	O	42	OD1	ASP	214	H-donor	3.41	−0.1	− 19.8089981
	C	22	OE1	GLU	276	H-donor	3.45	−0.5	
	O	27	OD2	ASP	408	H-donor	2.98	−1	
	C	31	OE1	GLN	181	H-donor	3.43	−0.1	
	O	40	O	TYR	71	H-donor	2.59	−1	
	O	44	OE2	GLU	276	H-donor	2.96	−0.6	
	O	53	OD2	ASP	349	H-donor	2.62	−3.6	
	C	64	O	VAL	303	H-donor	3.36	−0.1	
	O	21	CE2	PHE	300	H-acceptor	3.68	−0.1	
	O	42	NH2	ARG	212	H-acceptor	2.98	−1.6	
	O	44	CZ	PHE	300	H-acceptor	3.7	−0.1	
	O	53	NH1	ARG	439	H-acceptor	2.72	−0.7	
	O	55	CD	ARG	439	H-acceptor	3.14	−0.2	
	O	68	OH	TYR	344	H-acceptor	2.96	−0.5	
	−6	ring	CE1	PHE	158	pi-H	3.35	−0.2	
	−6	ring	CG	GLN	350	pi-H	3.54	−0.2	
	−6	ring	6-ring	PHE	177	pi-pi	3.13	0	
	−6	ring	6-ring	PHE	300	pi-pi	3.97	0	
15	O	23	OD2	ASP	68	H-donor	2.63	−7	−15.9700079
	O	25	O	ASP	349	H-donor	2.66	−4.3	
	O	14	NE2	HIS	348	H-acceptor	3.25	−0.1	
	O	19	ND2	ASN	347	H-acceptor	2.86	−3.7	
	O	23	NE2	HIS	111	H-acceptor	3.44	−0.2	
	−6	ring	CD	ARG	439	pi-H	4.04	−0.2	
16	O	24	OE1	GLN	181	H-donor	2.88	−2.5	−12.9178295
	O	23	NE2	HIS	111	H-acceptor	3.03	−0.7	
	O	23	CG2	THR	215	H-acceptor	3.53	−0.1	
	O	26	CE2	PHE	300	H-acceptor	4.05	−0.1	
	−6	ring	6-ring	PHE	177	pi-pi	3.8	0	
17	O	29	OD2	ASP	408	H-donor	2.75	−2.2	−17.0351429
	O	29	OD1	ASP	408	H-donor	3.44	−0.1	
	O	44	O	ASP	349	H-donor	2.97	−1.4	
	O	23	NE2	HIS	245	H-acceptor	3.22	−2.2	
	O	26	ND2	ASN	241	H-acceptor	2.93	−1	
	O	48	ND2	ASN	347	H-acceptor	2.93	−2.6	
	O	26	ND1	HIS	279	ionic	3.88	−0.8	
	O	26	NE2	HIS	279	ionic	3.7	−1.2	
	O	50	NH1	ARG	439	ionic	3.36	−2.5	
	O	48	6-ring	PHE	300	H-pi	3.32	−0.2	
	−6	ring	5-ring	HIS	279	pi-pi	3.91	0	
18	C	10	OD2	ASP	349	H-donor	3.45	−0.8	−13.8796387
	C	19	OD1	ASN	347	H-donor	3.6	−0.1	
	O	29	OE1	GLN	181	H-donor	2.79	−3.8	
	O	26	NH1	ARG	212	H-acceptor	3.2	−0.3	
	O	26	NH2	ARG	212	H-acceptor	2.87	−0.8	
	O	26	NE2	HIS	348	H-acceptor	3.34	−1.6	
	O	27	CG2	VAL	108	H-acceptor	3.54	−0.1	
	C	17	5-ring	HIS	348	H-pi	4.44	−1.4	
	−6	ring	NE2	HIS	111	pi-H	4.45	−0.1	
19	O	43	OD1	ASP	408	H-donor	3.32	−0.8	−16.4973335
	O	25	NE	ARG	312	ionic	3.65	−1.4	
	O	25	NH1	ARG	312	ionic	2.9	−5.1	
	C	21	6-ring	PHE	300	H-pi	3.73	−0.3	
	C	29	5-ring	HIS	279	H-pi	4.66	−0.2	
	−6	ring	CD	ARG	312	pi-H	3.45	−0.1	
20	C	29	OD2	ASP	408	H-donor	3.35	−0.8	− 18.9063892
	O	43	OD1	ASP	408	H-donor	3.63	−0.1	
	O	45	OD2	ASP	408	H-donor	3.02	−1.2	
	O	47	O	PHE	157	H-donor	3.13	−1.3	
	O	45	CE1	TYR	313	H-acceptor	3.37	−0.2	
	O	45	CD	ARG	439	H-acceptor	3.51	− 0.2	
	C	22	6-ring	PHE	157	H-pi	3.81	−0.8	
	−6	ring	NH1	ARG	439	pi-cation	4.19	−0.1	
	−6	ring	6-ring	PHE	177	pi-pi	3.94	0	
21	O	44	OE1	GLU	276	H-donor	2.98	−1.5	−17.2185078
	O	50	OE2	GLU	304	H-donor	2.7	−3.3	
	O	52	OD2	ASP	408	H-donor	2.83	−2.9	
	O	44	CG2	THR	215	H-acceptor	3.28	−0.1	
	O	44	CE2	PHE	300	H-acceptor	3.55	−0.1	
	−6	ring	N	ARG	312	pi-H	4.7	−0.2	
	−6	ring	CB	ARG	312	pi-H	3.76	−0.1	
	−6	ring	CD	ARG	312	pi-H	4.37	− 0.5	
22	C	20	OD2	ASP	408	H-donor	3.25	−0.6	−18.1408939
	C	22	OD1	ASN	347	H-donor	3.34	−0.3	
	O	32	OD2	ASP	408	H-donor	2.97	−4.7	
	O	46	OE2	GLU	276	H-donor	3.03	−0.6	
	O	38	NH2	ARG	212	H-acceptor	2.98	−0.4	
	O	38	NE2	HIS	348	H-acceptor	3.15	−5.8	
	O	44	ND2	ASN	347	H-acceptor	2.95	−2.2	
	O	38	NH1	ARG	212	ionic	3.6	−1.5	
	O	38	NH2	ARG	212	ionic	2.98	−4.6	
	C	6	6-ring	PHE	177	H-pi	3.96	−0.6	
	C	24	6-ring	PHE	300	H-pi	3.6	−0.1	
	C	39	5-ring	HIS	348	H-pi	3.98	−0.3	
	−6	ring	CB	PHE	157	pi-H	4.63	−0.4	
	−6	ring	CE1	PHE	177	pi-H	3.41	−0.4	
	−6	ring	NH2	ARG	212	pi-cation	4.71	−0.1	
23	O	46	OD2	ASP	68	H-donor	2.8	−1.8	−16.2608795
	O	48	OD2	ASP	68	H-donor	3.1	−2.6	
	O	52	OD1	ASP	214	H-donor	2.65	−2.4	
	C	55	OE2	GLU	276	H-donor	3.31	−0.3	
	O	14	CE2	PHE	300	H-acceptor	3.64	−0.1	
	O	46	CZ	PHE	158	H-acceptor	3.67	−0.1	
	O	46	NH1	ARG	439	H-acceptor	3.02	−2.6	
	O	46	NH2	ARG	439	H-acceptor	2.92	−2.8	
	O	52	CG2	THR	215	H-acceptor	3.78	−0.1	
	C	35	6-ring	PHE	177	H-pi	3.6	−0.3	
	C	55	6-ring	PHE	300	H-pi	4.77	− 0.1	
24	C	2	OE1	GLN	350	H-donor	3.49	−0.3	−18.0597305
	O	24	OD2	ASP	68	H-donor	3.06	−1.2	
	C	38	O	PHE	157	H-donor	3.67	−0.1	
	C	42	OD2	ASP	408	H-donor	3.11	−0.1	
	C	65	O	PRO	309	H-donor	3.57	−0.1	
	O	71	OD1	ASN	241	H-donor	3.05	−0.2	
	O	35	CE1	PHE	177	H-acceptor	3.79	−0.1	
	O	69	ND2	ASN	241	H-acceptor	3.04	−1.5	
	O	71	ND2	ASN	241	H-acceptor	2.93	−0.6	
	C	18	6-ring	PHE	177	H-pi	3.95	− 0.1	
	O	26	6-ring	PHE	177	H-pi	3.14	−0.1	
	C	44	6-ring	PHE	157	H-pi	4.48	−0.7	
	C	54	6-ring	PHE	157	H-pi	3.99	−0.2	
	−6	ring	NH1	ARG	439	pi-cation	3.66	−0.1

### Docking analysis of α-amylase

**Fig. 3:**
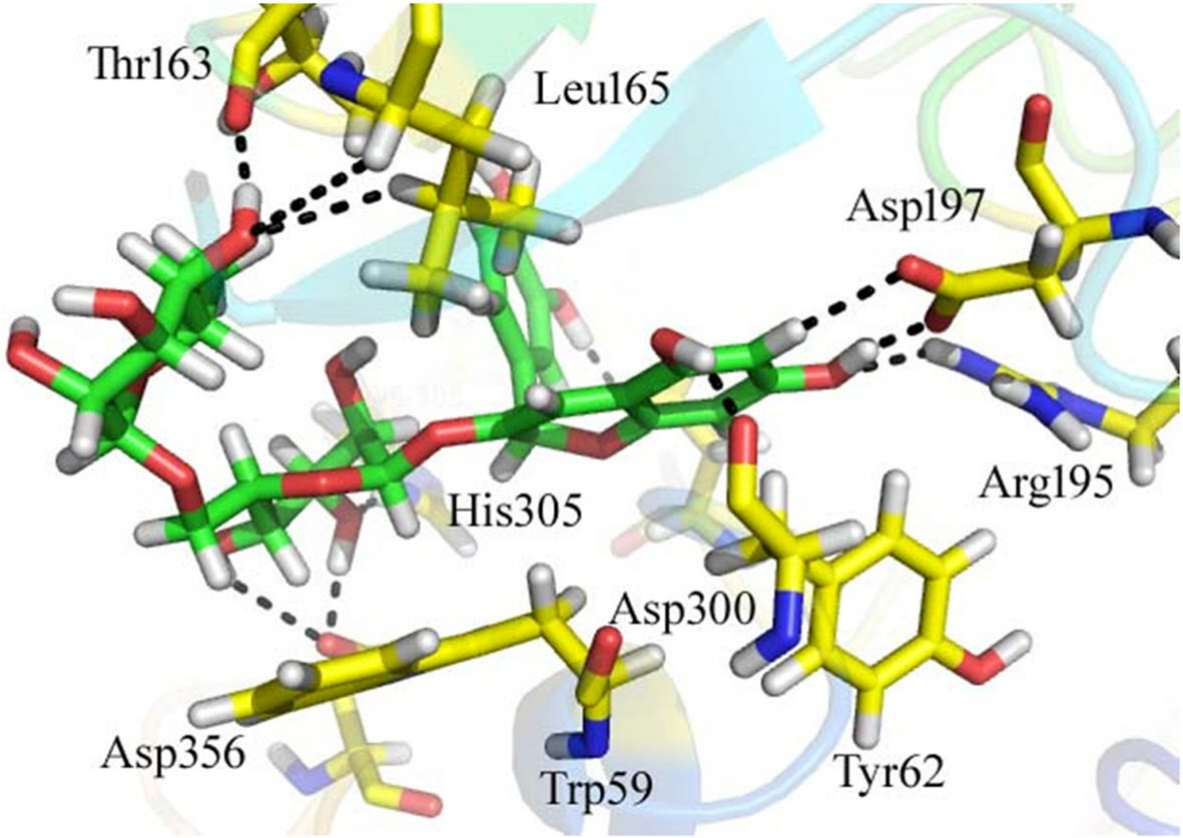
Docking conformation of compound **24** in the active site of *α*-amylase

Docking against revealed that the identified compounds were well accommodated in the active site residues (Trp 59, Tyr 62, Thr 163, Leu 165, Arg 195, Asp 197, Glu 240, Asp 300, His 305, Asp 356) of the target enzyme α-Amylase. From the docking conformation of the compounds, it was observed that compound 24 was the top active compound (docking score = − 15.03757). This compound formed 11 hydrogen bonds, three H-pi and one pi-H contacts with the active site residues of α-amylase (Fig. 3). The interactions detail of the compound is mentioned in Table 4. The inhibition of this compound might be due to the availability of the electron donating group (−OH) and electronic cloud system may be the reason of the excellent *in-silico* activity of the compound.

**Table 4:** Results of molecular docking studies with the identified compounds against *α*-amylase

S. No	Ligand		Receptor				Interaction	Distance	E (kcal/mol)	Docking score
1	O	16	NE2	HIS	299	(A)	H-acceptor	2.88	−3.7	−7.69861555
2	O	18	NH2	ARG	195	(A)	H-acceptor	2.92	−1.9	−8.24910164
	O	18	NE2	HIS	299	(A)	H-acceptor	3.45	−0.8	
	O	9	6-ring	TRP	58	(A)	H-pi	4.64	−0.7	
3	O	24	OH	TYR	151	(A)	H-acceptor	3.06	−0.3	−9.24960327
	O	24	NE2	HIS	201	(A)	H-acceptor	3.15	−1.1	
4	O	20	NE2	GLN	63	(A)	H-acceptor	3.1	−1.8	−7.81459236
	−6	ring	CZ3	TRP	58	(A)	pi-H	4.05	−0.1	
	−6	ring	CH2	TRP	58	(A)	pi-H	4.17	−0.1	
5	O	12	NE2	HIS	299	(A)	H-acceptor	3.11	−1.1	−8.02968025
	O	13	CZ3	TRP	58	(A)	H-acceptor	3.83	−0.1	
	O	13	NE2	HIS	299	(A)	H-acceptor	2.97	−6.9	
	O	12	NH2	ARG	195	(A)	ionic	3.23	−3.1	
6	O	18	NE2	HIS	101	(A)	H-acceptor	3.23	−3.3	−6.82410812
	O	19	NH2	ARG	195	(A)	H-acceptor	3.4	−1.1	
	O	19	NH2	ARG	195	(A)	ionic	3.4	−2.3	
7	O	18	O	TYR	62	(A)	H-donor	3.25	−0.5	−10.6273155
	O	22	O	TYR	62	(A)	H-donor	2.95	−0.9	
	O	16	CH2	TRP	58	(A)	H-acceptor	3.73	−0.1	
	−6	ring	CG2	THR	163	(A)	pi-H	3.86	−0.3	
8	O	17	OD1	ASP	197	(A)	H-donor	3.02	−3	−11.8703232
	O	39	OD1	ASP	356	(A)	H-donor	3.32	−0.3	
	O	16	NH2	ARG	195	(A)	H-acceptor	2.96	−1.7	
	O	16	N	ALA	198	(A)	H-acceptor	3.38	−0.1	
	O	19	CD1	LEU	165	(A)	H-acceptor	3.46	−0.1	
	O	36	NE2	GLN	63	(A)	H-acceptor	3.02	−1.3	
	O	41	CH2	TRP	58	(A)	H-acceptor	3.68	−0.1	
	O	16	NH2	ARG	195	(A)	ionic	2.96	−4.7	
	−6	ring	CG2	ILE	235	(A)	pi-H	4.07	−0.1	
	−6	ring	CD2	HIS	305	(A)	pi-H	3.44	−0.1	
	−6	ring	N	ALA	307	(A)	pi-H	4.28	−0.4	
	−6	ring	CB	ALA	307	(A)	pi-H	4.29	−0.3	
9	O	33	OD1	ASP	197	(A)	H-donor	3.05	−2.2	−9.00002575
	O	33	OD2	ASP	197	(A)	H-donor	3.11	−1.4	
	O	40	OD1	ASP	197	(A)	H-donor	2.83	−2.6	
	O	19	CB	TRP	59	(A)	H-acceptor	3.24	−0.1	
	O	33	NE2	HIS	101	(A)	H-acceptor	3.23	−0.7	
	O	40	NH2	ARG	195	(A)	H-acceptor	3.07	−0.2	
	O	40	CB	ALA	198	(A)	H-acceptor	3.5	−0.1	
	C	9	5-ring	TRP	59	(A)	H-pi	4.35	−0.1	
10	O	22	OE1	GLU	240	(A)	H-donor	3.38	−0.4	− 10.8587704
	O	22	OE2	GLU	240	(A)	H-donor	3.17	−0.2	
	O	40	OD1	ASP	197	(A)	H-donor	3.13	−2.5	
	O	22	CG	LEU	237	(A)	H-acceptor	3.48	−0.1	
	O	22	CD1	LEU	237	(A)	H-acceptor	3.47	−0.1	
	O	39	CG2	ILE	235	(A)	H-acceptor	3.78	−0.1	
	O	39	N	ALA	307	(A)	H-acceptor	2.97	−4.6	
	−6	ring	CB	ALA	198	(A)	pi-H	4.15	−0.5	
11	O	62	OD2	ASP	197	(A)	H-donor	3.12	−1.3	−10.0222139
	O	19	CG2	ILE	235	(A)	H-acceptor	3.47	−0.1	
	C	55	5-ring	HIS	101	(A)	H-pi	4.64	−0.5	
	C	60	6-ring	TRP	58	(A)	H-pi	4.77	−0.1	
	O	62	5-ring	HIS	101	(A)	H-pi	4.84	−0.1	
	−6	ring	CB	TYR	62	(A)	pi-H	3.74	−0.3	
12	O	41	O	THR	163	(A)	H-donor	2.98	−1.1	−7.80944586
13	O	23	NE2	GLN	63	(A)	H-acceptor	3	−2.5	−9.71675777
	O	45	NE2	HIS	299	(A)	H-acceptor	2.97	−6.2	
	O	44	NH2	ARG	195	(A)	ionic	3.99	−0.5	
	O	45	NH1	ARG	195	(A)	ionic	3.92	−0.7	
	O	45	NH2	ARG	195	(A)	ionic	2.95	−4.8	
14	O	25	OE1	GLU	233	(A)	H-donor	2.89	−2.8	−11.215291
	O	68	OD1	ASP	356	(A)	H-donor	3.05	−4	
	O	53	NE2	GLN	63	(A)	H-acceptor	2.98	−2.3	
15	O	22	OD1	ASP	197	(A)	H-donor	2.93	−2.3	−14.5967274
	O	20	CG	LEU	162	(A)	H-acceptor	3.69	−0.1	
	O	21	NH2	ARG	195	(A)	H-acceptor	3.11	−1.5	
	O	21	NE2	HIS	299	(A)	H-acceptor	3.61	−1	
	O	21	NH2	ARG	195	(A)	ionic	3.11	−3.8	
	−6	ring	CB	ALA	198	(A)	pi-H	4.48	−0.2	
	−6	ring	CG2	ILE	235	(A)	pi-H	4.56	−0.3	
	−6	ring	CD1	ILE	235	(A)	pi-H	3.76	−0.1	
16	C	7	OD1	ASP	300	(A)	H-donor	3.59	−0.1	−11.8797693
	C	7	OD2	ASP	300	(A)	H-donor	3.53	−0.1	
	O	24	OE1	GLU	233	(A)	H-donor	3.73	−0.1	
	O	23	NH2	ARG	195	(A)	H-acceptor	2.94	−1.7	
	O	23	NE2	HIS	299	(A)	H-acceptor	3.15	−2.3	
	O	31	CB	TYR	62	(A)	H-acceptor	3.45	−0.1	
	O	23	NH1	ARG	195	(A)	ionic	3.79	−1	
	O	23	NH2	ARG	195	(A)	ionic	2.94	−4.9	
	C	11	6-ring	TRP	58	(A)	H-pi	4.83	−0.4	
	C	14	5-ring	TRP	59	(A)	H-pi	4.38	−0.2	
	O	27	5-ring	TRP	59	(A)	H-pi	3.66	−2.4	
	−6	ring	NE2	GLN	63	(A)	pi-H	3.67	−0.1	
	−6	ring	CD1	LEU	165	(A)	pi-H	4.89	−0.3	
17	O	24	O	TYR	62	(A)	H-donor	3.05	−2.1	−10.7860909
	C	33	O	HIS	305	(A)	H-donor	3.51	−0.2	
	C	39	O	HIS	305	(A)	H-donor	3.49	−0.2	
	O	26	NE2	GLN	63	(A)	H-acceptor	2.98	−5.6	
	O	26	CD1	LEU	165	(A)	H-acceptor	3.97	−0.1	
	−6	ring	CB	TYR	62	(A)	pi-H	4.83	−0.1	
	−6	ring	CD1	LEU	165	(A)	pi-H	4	−0.2	
	−6	ring	CG2	ILE	235	(A)	pi-H	4.31	−0.1	
18	O	27	O	TYR	62	(A)	H-donor	3.06	−1	−7.62582397
19	C	18	O	HIS	305	(A)	H-donor	3.65	−0.2	−11.0418396
	O	25	NE2	HIS	305	(A)	H-acceptor	3.04	−2.1	
	C	29	5-ring	HIS	305	(A)	H-pi	4.89	−0.1	
	− 6	ring	CH2	TRP	58	(A)	pi-H	4.87	−0.1	
	−6	ring	CA	GLY	306	(A)	pi-H	3.75	−0.1	
	−6	ring	CB	ALA	307	(A)	pi-H	3.65	−0.4	
20	O	53	OD2	ASP	356	(A)	H-donor	3.19	−1.3	−12.1673326
	O	24	NE2	HIS	305	(A)	H-acceptor	3.04	−1.9	
	O	43	CD1	LEU	165	(A)	H-acceptor	3.94	−0.1	
	O	45	NE2	GLN	63	(A)	H-acceptor	3.07	−0.5	
	C	40	5-ring	TRP	59	(A)	H-pi	4.1	−0.1	
	O	45	5-ring	TRP	59	(A)	H-pi	3.98	−0.5	
	O	45	6-ring	TRP	59	(A)	H-pi	4.8	−0.2	
21	C	1	OD1	ASP	197	(A)	H-donor	3.25	−0.1	−12.8208132
	O	34	OD1	ASP	197	(A)	H-donor	2.92	−3.6	
	O	42	OH	TYR	151	(A)	H-acceptor	2.9	−1.1	
	O	42	CD1	LEU	162	(A)	H-acceptor	3.77	−0.1	
	O	44	CA	GLY	306	(A)	H-acceptor	3.39	−0.1	
	O	44	N	ALA	307	(A)	H-acceptor	2.96	−2.2	
	−6	ring	CB	ALA	198	(A)	pi-H	4.1	−0.8	
22	C	28	OD1	ASP	300	(A)	H-donor	3.13	−0.5	− 10.3951654
	O	36	O	TYR	62	(A)	H-donor	2.83	−1.5	
	O	52	OD1	ASP	300	(A)	H-donor	3.36	−0.1	
	O	31	CZ3	TRP	58	(A)	H-acceptor	3.55	−0.1	
23	O	23	OD1	ASP	197	(A)	H-donor	2.9	−0.5	−13.9205046
	C	60	OD2	ASP	300	(A)	H-donor	3.45	−0.2	
	O	46	NE2	HIS	305	(A)	H-acceptor	3.44	−0.6	
	O	50	NE2	HIS	305	(A)	H-acceptor	3.2	−0.5	
	O	52	CD2	LEU	165	(A)	H-acceptor	3.7	−0.1	
	−6	ring	CB	ALA	198	(A)	pi-H	4.43	−0.2	
	−6	ring	CB	ALA	307	(A)	pi-H	4.85	−0.1	
24	C	2	OD2	ASP	197	(A)	H-donor	3.47	−0.5	−15.0375738
	O	26	OD1	ASP	300	(A)	H-donor	2.55	−3	
	O	28	OD1	ASP	197	(A)	H-donor	2.5	−3.6	
	O	30	O	TYR	62	(A)	H-donor	2.64	−2.2	
	O	47	OD1	ASP	356	(A)	H-donor	2.64	−1.8	
	O	49	OD1	ASP	356	(A)	H-donor	3.36	−0.3	
	O	73	O	THR	163	(A)	H-donor	2.72	−2.2	
	O	28	NH2	ARG	195	(A)	H-acceptor	3.08	−0.1	
	O	47	NE2	HIS	305	(A)	H-acceptor	3	−2.9	
	O	73	CG	LEU	165	(A)	H-acceptor	3.68	−0.1	
	O	73	CD2	LEU	165	(A)	H-acceptor	3.5	−0.1	
	C	33	5-ring	TRP	59	(A)	H-pi	3.59	−1	
	C	38	5-ring	TRP	59	(A)	H-pi	4.09	−0.2	
	C	44	6-ring	TRP	59	(A)	H-pi	3.86	−0.2	
	−6	ring	CB	TYR	62	(A)	pi-H	3.68	−0.1	

### Nutritional studies

In preliminary nutritional analysis of crude powder, 17.38% proteins contents, 15.70% moisture content, 10.73% ash content and 4.18% fat content as summarized in Table 5. These presence of these food contents signify the nutritional potentials of the plant. The plant is used as tea decoction in some countries and is used as salad. Nutritional finding suggests that the plant might be a useful source for the dietary management of proteins and fats. Further, due to the moisture contents the powder materials may need proper storage to avoid fungi growth and deterioration [72].

**Table 5:** Nutritional contents of *P. hydropiper* crude powder

**S. No**	**Proteins % Contents**					
	**Weight of sample**	**Vol. of titer**	**Bulk**	**Titer - bulk**	**N%**	**Protein %**
1	0.552	23.8	3.3	20.5	2.859601	**17.87251**
2	0.6028	25	3.3	21.7	2.771898	**17.32436**
3	0.6042	24.6	3.3	21.3	2.714499	**16.96562**
**% Moisture Contents**						
**S. No**	**Empty Dish weight**	**Sample + Dish**	**Sample weight**	**After heating**	**Moisture weight**	**% Moisture**
1	16.3058	18.24	1.9342	17.9325	0.3075	**15.89805**
2	16.3003	18.4246	2.1243	18.0682	0.3564	**16.77729**
3	14.6234	15.841	1.2176	15.6652	0.1758	**14.43824**
**% Ash Contents**						
**S. No**	**Empty dish wt**	**Sample + dish wt.**	**Sample wt.**	**Wt. after heating**	**Ash wt.**	**% ash**
1	23.1155	24.1033	0.9878	23.2244	0.1089	**11.0245**
2	20.9005	22.1234	1.2229	21.0294	0.1289	**10.54052**
3	29.3161	31.2188	1.9027	29.5186	0.2025	**10.64277**
**% Fat Contents**						
**S. No**	**Sample weight**	**Empty bk. wt**	**BK + Oil Wt.**	**Oil Wt.**	**% Fat**	
1	2.2468	29.6871	29.7832	0.0961	**4.277194**	–
2	2.6881	22.654	22.7482	0.0942	**3.504334**	–
3	1.9867	28.3641	28.4588	0.0947	**4.766699**	–

### Toxicological assessments

Acute toxicity studies reveled no lethality in animal groups as well as no abnormal behavioral changes in animals up to 24 h of samples administration. In this study Ph.Cr and Ph.Hex were found most effective against different blood groups. Haemagglutination activity of Ph.Cr was most prominent (+++) against AB^+^, AB^−^, O^+^ and O^−^ blood groups. Ph.Hex was highly effective against A^+^, A^−^ and B^+^ blood groups at 1:1 concentration (Table 6).

**Table 6:** Result of hemagglutination effect of *P. hydropiper* extracts and saponins on different blood groups at different concentrations

Blood groups	Ph.Cr			Ph.Hex			Ph.Chf			Ph.EtAc			Ph.Bt			Ph.Aq		
	1:1	1:2	1:4	1:1	1:2	1:4	1:1	1:2	1:4	1:1	1:2	1:4	1:1	1:2	1:4	1:1	1:2	1:4
A+	–	–	–	+++	++	+	–	–	–	–	–	–	–	–	–	–	–	–
A-	++	+	+	+++	++	++	–	–	–	+	+	–	–	–	–	+	+	+
B+	–	–	–	+++	+++	+++	–	–	–	–	–	–	–	–	–	+	+	+
B-	–	–	–	–	–	–	–	–	–	–	–	–	–	–	–	–	–	–
AB+	+++	++	++	+++	++	++	–	–	–	++	+	–	+++	++	++	–	–	–
AB-	+++	++	++	+++	++	++	++	++	+	++	+	+	+++	++	++	–	–	–
O+	+++	++	++	+++	++	++	–	–	–	–	–	–	+++	++	++	–	+	+
O-	+++	++	+	–	–	–	–	–	–	–	–	–	–	–	–	–	–	–

**+++:** High hemagglutination activity, **++:** Intermediate activity, **+:** Low activity and **- =** No activity

Plant agglutinins, also called phytohemagglutinins, cause haemagglutination of human and animal erythrocytes (RBCs). These phyto-hemagglutinins/phytolectins have wide range of applications as research tools in diverse biological activities like mitogenic action, cancer chemotherapy and cell membrane structure analysis [73]. These are also utilized as a drug targets, separation and characterization of glycoconjugates, glycopeptides, in histochemistry and cell differentiations techniques [74, 75]. Traditionally *P. hydropiper* is used in bleeding disorders and to repair ruptured blood vessels [76].

## Conclusions

This study revealed that *P. hydropiper*, exhibit considerable amount of important secondary metabolites which might contribute to the α-glucosidase and α-amylase inhibition potentials of the plant. The same was confirmed by molecular simulation studies performed on identified compounds against these enzymes. Plant has significant proteins, fat contents, could be a good source of important valuable plant lectins which justify its ethnomedicinal uses in bleeding disorders and is safe at the test concentrations in animals. Further in-vivo anti-diabetic studies are required for potential uses of the plant in type-2 diabetes.

## Data Availability

Data related to the current paper can be provided upon request to the corresponding author.
